# Rice seeds biofortification using biogenic ıron oxide nanoparticles synthesized by using *Glycyrrhiza glabra*: a study on growth and yield ımprovement

**DOI:** 10.1038/s41598-024-62907-1

**Published:** 2024-05-29

**Authors:** Sidra Ahmad, Nayab Ahmad, Md. Shahinoor Islam, Mian Afaq Ahmad, Sezai Ercisli, Riaz Ullah, Ahmed Bari, Iqbal Munir

**Affiliations:** 1 Institute of Biotechnology and Genetic Engineering, The University of Agriculture, Peshawar, Pakistan; 2 Department of Chemical Engineering, Bangladesh University of Engineering and Technology, Dhaka, 1000 Bangladesh; 3 Department of Textile Engineering, Daffodil International University, Dhaka, 1341 Bangladesh; 4 Department of Horticulture, Faculty of Agriculture, Ataturk University, 25240 Erzurum, Turkey; 5 Department of Pharmacognosy College of Pharmacy, King Saud University, Riyadh, Saudi Arabia; 6 Department of Pharmaceutical Chemistry, College of Pharmacy, King Saud University, Riyadh, Saudi Arabia

**Keywords:** *Glycyrrhiza glabra*, Iron oxide nanoparticles, Nanopriming of rice seeds, Biofortification, Chlorophyll, Iron content, Plant sciences, Nanobiotechnology

## Abstract

Iron, a crucial micronutrient, is an integral element of biotic vitality. The scarcity of iron in the soil creates agronomic challenges and has a detrimental impact on crop vigour and chlorophyll formation. Utilizing iron oxide nanoparticles (IONPs) via nanopriming emerges as an innovative method to enhance agricultural efficiency and crop health. The objective of this study was to synthesize biogenic IONPs from *Glycyrrhiza glabra* (*G. glabra*) plant extract using green chemistry and to evaluate their nanopriming effects on rice seed iron levels and growth. The synthesized IONPs were analyzed using UV–Vis spectroscopy, Fourier-transform infrared spectroscopy (FTIR), Scanning electron microscope (SEM), Transmission electron microscopy (TEM), and Energy-dispersive X-ray (EDX) techniques. The UV–Vis peak at 280 nm revealed the formation of IONPs. SEM and TEM showed that the nanoparticles were spherical and had an average diameter of 23.8 nm. Nanopriming resulted in a substantial enhancement in growth, as seen by a 9.25% and 22.8% increase in shoot lengths for the 50 ppm and 100 ppm treatments, respectively. The yield metrics showed a positive correlation with the concentrations of IONPs. The 1000-grain weight and spike length observed a maximum increase of 193.75% and 97.73%, respectively, at the highest concentration of IONPs. The study indicates that *G. glabra* synthesized IONPs as a nanopriming agent significantly increased rice seeds' growth and iron content. This suggests that there is a relationship between the dosage of IONPs and their potential for improving agricultural biofortification.

## Introduction

Iron is an important micronutrient for plants, as it is involved in numerous physiological and biochemical processes^1^. It is an essential part of enzymes that are involved in photosynthesis, respiration, and nitrogen fixation^2^. Synthesis of chlorophyll^3^, DNA, RNA, and proteins, required for plant growth and development, is greatly boosted by the presence of iron^1,4^. Deficiency of iron in plants can cause chlorosis, stunted growth, and low yields from agriculture and thus, maintaining an appropriate iron supply is critical for supporting healthy and fruitful growth of crops^5^.

In recent years, the use of eco-friendly and sustainable methods for the synthesis of nanoparticles has gained significant attention in various fields, including agriculture^6–8^. Green synthesis of nanoparticles is not only an easy method but also eco-friendly and cost-effective^9^, Various types of nanoparticles were made previously from different metal and metal oxides by the green synthesis method including, zinc and zinc oxide nanoparticles, iron and iron oxide nanoparticles, copper and copper oxide nanoparticles^10,11^. All these nanoparticles are synthesized by using plants or microorganisms which convert metal salts into their respective nanoparticles^12^. Among all biological methods, plant mediated synthesis is preferred^13^. Iron oxide nanoparticles (IONPs) have demonstrated remarkable promise among nanoparticles due to their unique physicochemical features and variety of uses^14^. Among IONPs, magnetite (Fe_3_O_4_) and maghemite (γ-Fe_2_O_3_) have gained considerable attention due to their unique magnetic and catalytic properties^15,16^. The interaction between IONPs and plants is complex, with various parameters influencing it, including nanoparticle concentration, size, surface charge, and coating materials^17^. Studies have reported that IONPs can be internalized by plants and translocated to various tissues, affecting plant physiology and biochemical processes and growth^18,19^. Furthermore, the distinctive physicochemical properties of IONPs, such as high surface area and redox activity, enable them to act as carriers for delivering nutrients, pesticides, or growth regulators to plants, thereby enhancing nutrient uptake, stress tolerance, and overall plant growth^18,20^.

There are a variety of ways to manufacture nanoparticles, such as chemical, physical, and eco-friendly procedures. Several physical techniques are known that break down bulk materials into nanoscale particles mechanically including ball milling and laser ablation^21^. Chemical techniques provide reasonable control over the size and structure of the nanoparticles by precipitating or chemically reducing metal salts^22^. The green synthesis technique of IONPs reduces the use of hazardous chemicals by producing nanoparticles from natural and ecologically favourable sources, such as plants or microbes^23,24^ and has emerged as a viable alternative to conventional procedures, where toxic chemicals and significant energy usage are required^25,26^. Nanoparticles can also be synthesized from algal extract^27^. Natural resources such as plants, microorganisms, or bio-waste are used as reducing or stabilizing agents in the production of nanoparticles^28,29^. This approach offers several advantages, including cost-effectiveness, low toxicity, and compatibility with sustainable development goals^30,31^. Researchers can not only reduce environmental effects by employing green synthesis methods, but they may also produce nanoparticles with regulated size, shape, and surface qualities that can be tuned for specific uses^32^.

Biofortification is a revolutionary agricultural strategy that aims to improve the nutritional content of major crops like rice, wheat, maize, and beans^33^. Biofortification addresses the widespread problem of hidden hunger or micronutrient deficiencies, which affect millions of people worldwide, particularly in developing regions that rely heavily on these crops for sustenance^33,34^. Biofortified crops provide a direct and accessible source of vital nutrients such as iron, zinc, and vitamin A, reducing the reliance on dietary supplements and diversifying diets for vulnerable populations, making them a sustainable and cost-effective solution to combat malnutrition and improve public health^35,36^.

The application of iron oxide nanoparticles for seed nano priming has emerged as a viable strategy in agricultural biotechnology, demonstrating substantial enhancements in several crop species. A study indicated that nanopriming pepper seeds with iron oxide nanoparticles resulted in improved root and vegetative growth, surpassing typical priming approaches^37^. Moreover, the use of these nanoparticles in wheat seeds resulted in heightened growth, photosynthesis, and improved antioxidant activity, demonstrating a favourable influence on the plant's ability to withstand stress and promote productivity^8,38^.

Licorice is a plant known by the botanical name Glycyrrhiza and is best known for its sweet-tasting roots. The sweet flavour of liquorice is derived from glycyrrhizin, a chemical found in the plant's root^39^. Licorice includes a variety of phytochemicals, including glycyrrhizin, which gives it its sweet flavour but can cause high blood pressure if consumed in excess^40^. Licorice also contains flavonoids and triterpenoids, which may have antioxidant and anti-inflammatory properties^41^. These phytochemicals are frequently used in traditional medicine for therapeutic purposes such as sore throat relief and digestive disorders^42,43^. The flavonoids present in liquorice have powerful antioxidant effects. These antioxidants are critical in reducing oxidative stress^44^.

Rice (*Oryza sativa*) is one of the most important staple crops worldwide and an essential component for fighting against world hunger and malnutrition since it is a significant source of carbohydrates and essential nutrients for energy for billions of people^45^. Therefore, increasing the yield of rice is essential for ensuring food security^46,47^. It has dietary fibre, several vitamins and minerals, including B vitamins and essential minerals, and a moderate quantity of protein^48^. Different varieties of rice have different nutritional values. For instance, brown rice with low fat retains its bran layers has more fibre and antioxidants than other varieties^49^. Typically, rice has a moderate amount of iron, which depends on the variety, growth environment, and processing techniques^50^.

Nanopriming to seeds is applied to promote germination, growth, and yield of crops^51,52^. Green synthesis in nanopriming, especially with IONPs made from licorice extract boosts plant iron content, and thus, received attention for sustainable agriculture. This improvement is crucial for rice cultivation, which is vital to global nutrition and can address micronutrient deficits, especially in rice-consuming communities. The use of licorice-extract-synthesized iron oxide nanoparticles in nanopriming could increase rice yield and iron content. Some previous literature shows that nanopriming by iron oxide nanoparticles can improve the iron content of wheat^53^ and rice^54^. However, this area needs extensive research and very limited literature is available on iron biofortification of crops by nanopriming using biologically synthesized nanoparticles. To the best of our knowledge, it is the first study in which nanoparticles prepared by liqorice extract are used as nanopriming agent to enhance iron content in rice seeds. Therefore, in this study, iron oxide nanoparticles were synthesized using liquorice extract and examined via UV–Vis spectroscopy, Fourier-transform infrared spectroscopy (FTIR), Scanning electron microscope (SEM), Transmission electron microscopy (TEM), and Energy-dispersive X-ray (EDX) techniques. Rice seeds nanoprimed with different concentrations of iron oxide nanoparticles were then grown in a greenhouse till maturity, and growth parameters, yield components and iron content in rice seeds were analyzed.

## Materials and methods

### Materials and reagents

Deionized water, pH meter, iron sulphate heptahydrate, UV-1700 PharmaSpec, XRD analysis equipment (JEOL Model JDX 3532), FT-IR analysis equipment (FTIR Model PerkinElmer UATR), SEM (JEOL model JSM-IT100), EDX (UK Model INCA 200), TEM, ethanol, distilled water, liquid nitrogen, 80% acetone, spectrophotometer, AAS (Atomic absorption spectroscopy, PerkinElmer S.No 80155070601), nitric acid, perchloric acid. The rice seeds were obtained from NIGAB (National Institute For Genomics & Advanced Biotechnology), while the plant specimens were sourced from the local herbarium.

### Ethical guidelines for plant studies

*Glycyrrhiza glabra* (cultivated variety) utilized in this study was purchased from the local herbarium and identified by a botanist. All procedures related to the acquisition and cultivation of *G. glabra* were conducted in accordance with relevant institutional, national, and international guidelines and legislation.

### Plant extract formation and synthesis of nanoparticles

The *G. glabra* herb was purchased, rinsed with distilled water, dried in the shade, and pulverized into a fine powder. About 10 g of plant powder was dissolved in 100 ml of distilled water and incubated for 60 min at 80 °C in a water bath. The pH of the aqueous extract was measured with a pH meter after filtration. 6 g of precursor salt iron sulphate heptahydrate was dissolved in 100 ml of aqueous plant extract solution and heated for 2 h at 80 °C. The sample was then centrifuged for 15 min at 10,000 rpm. The pellet that resulted was oven-dried and finely crushed to get fine nanopowders. This nanopowder was annealed in a furnace at 150 °C. Before and after the addition of the precursor salt, the pH of the solution was measured^55^.

### Characterization of iron oxide NPs

Iron oxide nanoparticles were dissolved in deionized water in a 1:1 ratio, and spectrum scans were made using a UV-1700 PharmaSpec in the wavelength range of 350–450 nm. XRD analysis (JEOL Model JDX 3532) was used to determine the crystalline form and average size of the synthesized nanoparticles. FT-IR analysis (FTIR Model PerkinElmer UATR) was performed to produce an infrared absorption and emission spectrum in order to identify functional groups and distinct phytochemical ingredients involved in the stabilization and reduction of nanoparticles. The morphology of biosynthesized zinc oxide nanoparticles was determined using an SEM (JEOL model JSM-IT100). EDX (UK Model INCA 200) was used to determine the elemental composition of the synthesized nanoparticles. TEM (JEOL Japan Model JEM-2100) analysis was done to find out the size and shape of nanoparticles.

### Plant growth and nanoparticles application

Rice seeds (FM variety) surface was appropriately sterilized with ethanol followed by distilled water wash. Different concentrations of iron oxide nanoparticle solutions were prepared, i.e. 25, 50 and 100 ppm. The seeds were soaked in these solutions for an hour with constant shaking. After an hour, seeds were rinsed with distilled water, dried and sown in pots in the greenhouse. Control seeds were primed with distilled water. Treatments included control 50 ppm, 100 ppm and 150 ppm of iron oxide nanoparticles. Initially, three seeds were sown in each pot. After one month, one healthy seedling was maintained in one pot. The experiment was performed with three replicates for each treatment in a complete randomized design. Plants were grown till harvesting after 120 days of sowing. At harvesting, root and shoot length and fresh and dry weights of roots and shoots were measured.

### Measurement of chlorophyll content

Chlorophyll contents were analyzed using the modified protocol^56^. Leaf tissues from all samples were taken after 80 days and crushed using liquid nitrogen. Then, 5 ml of 80% acetone was added to each sample. The extract was centrifuged at 300*g* for 5 min. The supernatant was taken from each sample by spectrophotometer to check absorbance at wavelengths 663 and 646 nm.

Chlorophyll a and b contents were measured using a formula.

$${\text{Chlorophyll}}\, {\text{a}} \left( {{\text{mg}}/{\text{g}}} \right) \, = { 12}.{25} \times {\text{A663}} - {2}.{79} \times {\text{A646}}$$

$${\text{Chlorophyll}}\, {\text{b}} \left( {{\text{mg}}/{\text{g}}} \right) \, = { 21}.{5}0 \times {\text{A646}} - {5}.{1}0 \times {\text{A663}}$$

### Measurement of grain yield and yield components

A random sample was collected from three pots at the stage of full growth in order to record the number of spikes, the length of the spikes, the number of tillers, the yield of grains, and the weight of one thousand grains.

### Determination of iron content in rice seeds

**Figure 1:**
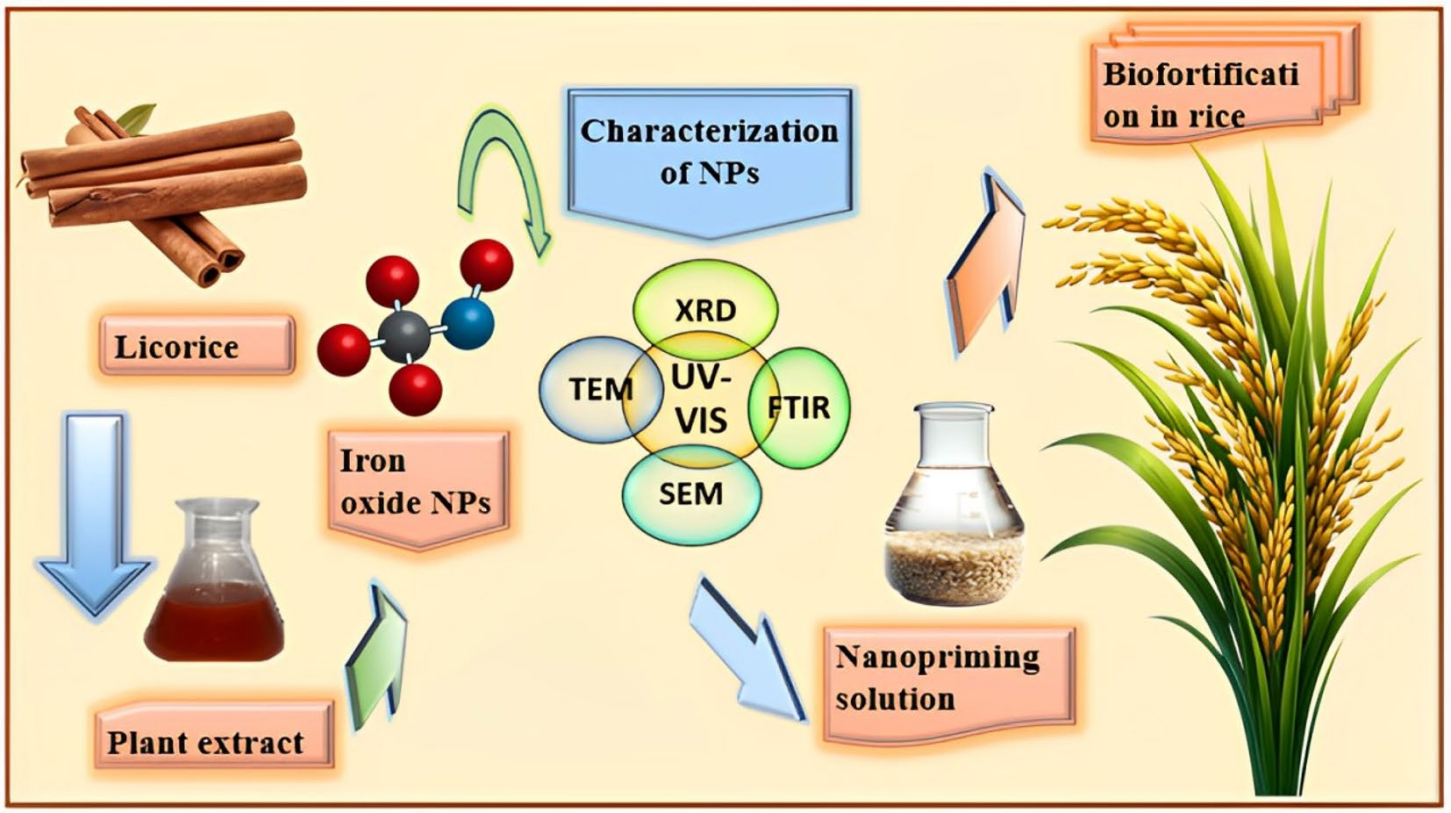
An overview of the methodology from licorice extract preparation to biofortification in rice by iron oxide nanoparticles.

Iron concentration in rice seeds was analyzed using AAS (PerkinElmer S.No 80155070601). About 0.5 g of rice powder was digested with nitric acid and perchloric acid in a ratio of 2:1. After 24 h, the mixture was heated till the appearance of white fumes. Then, it was filtered, and iron content was measured by AAS^57^. An overview of the methodology section is shown in Fig. 1.

### Statistical analysis

Data analysis was performed with IBM SPSS software. For each treatment, a one-way ANOVA was run with three replicates, and the Tukey HSD test was subsequently run. A p-value of less than 0.05 was considered significant.

## Results

### Characterization of iron oxide NPs

**Figure 2:**
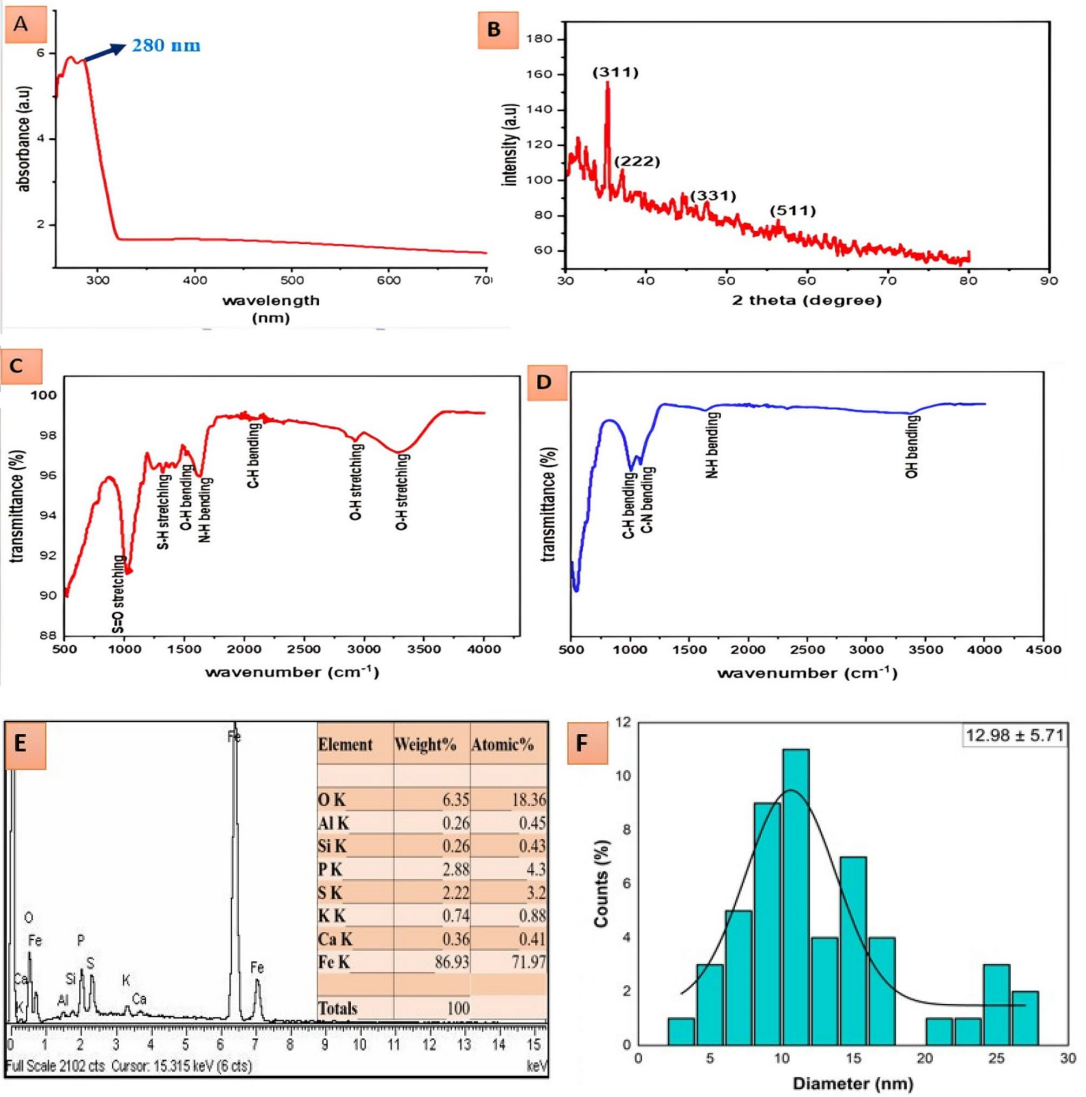
(**a**) UV–Vis spectrum of iron oxide nanoparticles. (**b**) XRD analysis of IONPs. (**c**) FTIR spectra of liquorice extract. (**d**) FTIR spectra of iron oxide nanoparticles. (**e**) EDX spectra and elemental composıtıon of iron oxide nanoparticles and (**f**) Size distribution of IONPs calculated by TEM.

UV spectra showed a characteristic peak at 280 nm, which confirms iron oxide nanoparticles (Fig. 2A). The X-ray diffraction (XRD) study showed clear peaks (Fig. 2B) at 2θ angles of 35.18°, 37.5°, 47.5°, and 56.24°, which corresponded to the Miller indices (311), (222), (331), and (511), respectively. The observed peaks in the iron oxide nanoparticles indicated the existence of crystallographic planes, which are indicative of a spinel structure. This structure is consistent with either magnetite (Fe_3_O_4_) phases, as specified by the JCPDS standards (JCPD files no.98-0073). The Debye–Scherrer equation was employed to compute the crystallite size based on the width of peaks. The resulting value was around 35.75 nm, suggesting the successful synthesis of nanoparticles.

**Figure 3:**
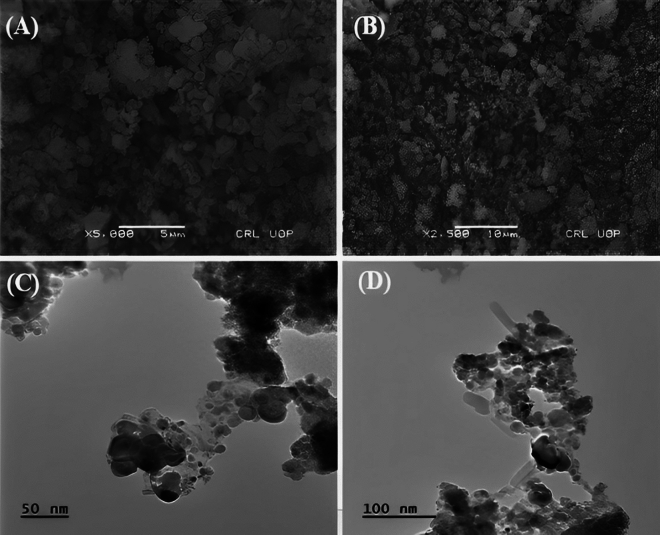
(**a**) SEM image of iron oxide nanoparticles (scale bar, 5 nm), (**b**) SEM image of iron oxide nanoparticles (scale bar, 10 nm), (**c**) TEM image of iron oxide nanoparticles (scale bar, 50 nm) and (**d**) TEM image of iron oxide nanoparticles (scale bar, 100 nm).

FTIR analysis was performed to identify the functional groups in plant extracts, which were responsible for the formation and stabilization of the nanoparticles. FTIR spectra of plant extract (Fig. 2C) showed IR bands at 1033 cm^−1^, 1315 cm^−1^, 1424 cm^−1^, 1627 cm^−1^, 2042 cm^−1^, 2920 cm^−1^ and 3272 cm^−1^, showing the presence of S=O stretching, S–H stretching, O–H bending, N–H bending, C–H bending, and O–H stretching, respectively (Table 1). FTIR spectra of iron oxide nanoparticles (Fig. 2D) showed IR bands at 1002 cm^−1^, 1087 cm^−1^, 1630 cm^−1^ and 3361 cm^−1^, which represent the presence of C–H bending, C–N bending, N–H bending and O–H stretching, respectively (Table 2). When FTIR spectra of plant extract were compared with FTIR spectra of nanoparticles, band shift was detected in the N–H bending and O–H stretching groups, which gives a clue that alcoholic and phenolic groups were involved in the synthesis process. This band shift confirmed the synthesis of iron oxide nanoparticles. EDX spectra confirmed the presence of iron and oxygen in higher concentrations (Fig. 2E). The amount of iron detected by EDX was 47.14%, and oxygen was 16% in the iron oxide nanoparticles sample (Fig. 2E). Size distribution of IONPs was described using histogram (Fig. 2F). SEM showed spherical and irregular shape nanoparticles while TEM images showed the spherical morphology of synthesized iron oxide nanoparticles but some of them were rod in shape (Fig. 3A–D). Both SEM and TEM images confirm agglomerated nanoparticles of iron oxide. The average diameter of the nanoparticles calculated by TEM images was 12.98 ± 5.71 nm.

**Table 1:** Different functional groups present in FTIR spectra of IONPs with their corresponding compounds.

Frequency	Functional group	Compound	References
1033	S=O stretching	Glucosinolates	^58,59^
1315	S–H stretching	Glucosinolates	^59,60^
1424	O–H bending	Alcohol or phenol	^61^
1627	N–H bending	Amine	^62^
2042	C–H bending	Alkyn	^63^
2920	O–H stretching	Alcohol	^64^
3272	O–H stretching	Carboxylic acid	^61^

**Table 2:** Different functional groups present in FTIR spectra of *G. glabra* plant extract with their corresponding compounds.

Frequency	Functional group	Compound	References
1002	C–H bending	Triterpenoids	^63,65^
1087	C–H bending	Triterpenoids	^65,66^
1630	O–H bending	Terpenoids and phenolics	^62^
3361	O–H bending	Alcohol	^67^

### Effect of NPs on plant growth

**Figure 4:**
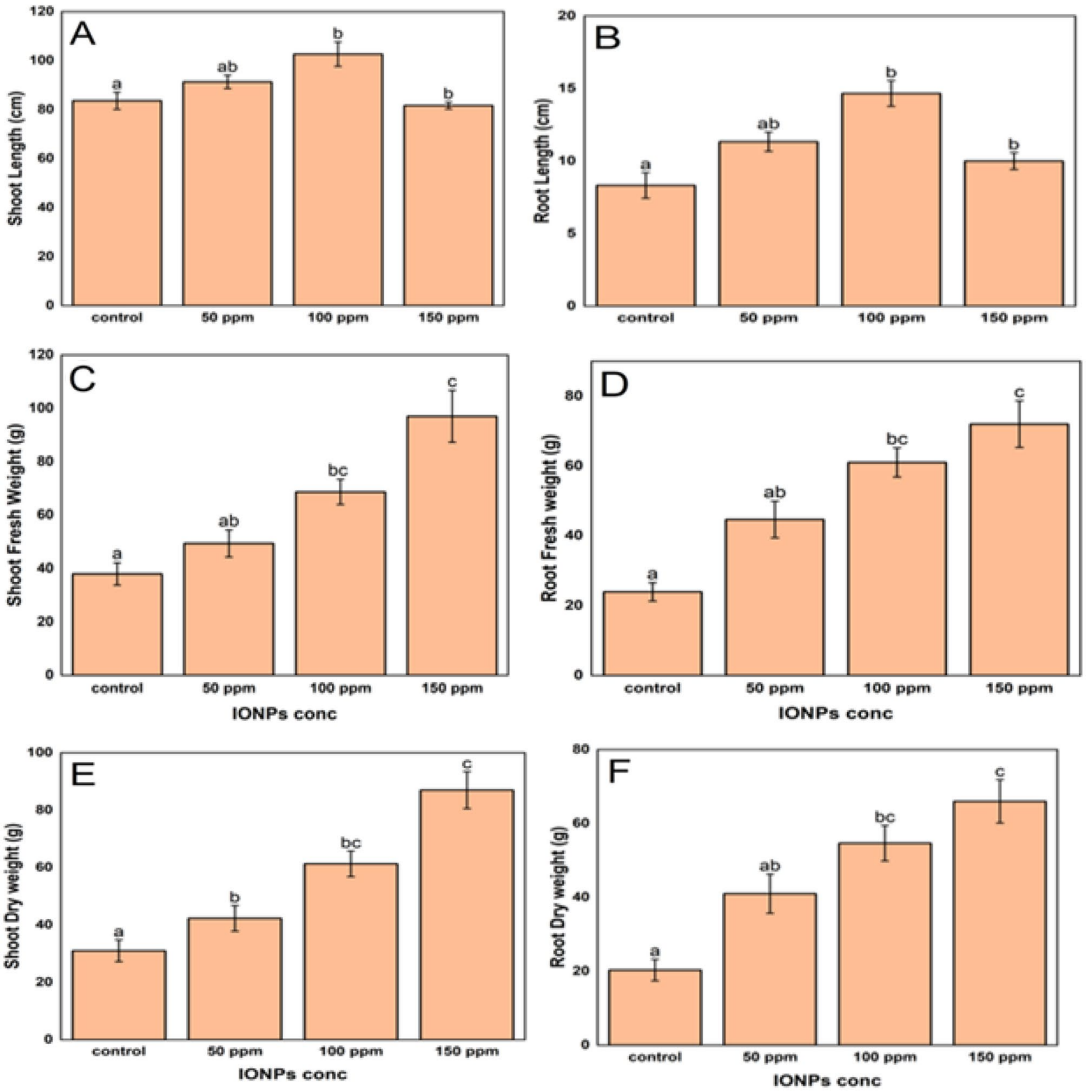
Effect of iron oxide nanoparticles on plant growth parameters. (**a**) shoot length (cm), (**b**) root length (cm), (**c**) shoot fresh weight (g), (**d**) root fresh weight (g), (**e**) shoot dry weight (g) and (**f**) root dry weight (g). Different symbols signify significant differences at p ≤ 0.05 between distinct treatments. Values are means of three replicates.

Shoot lengths of plants germinated from seeds treated with IONP concentrations of 50 ppm and 100 ppm were found to be greater than those of the control group, as shown in Fig. 4A. The shoot lengths of rice plants increased by 9.25% and 22.8% at 50 ppm and 100 ppm, respectively, compared to the control. In the same manner, the root lengths of all plants subjected to different treatments (50 ppm, 100 ppm, and 150 ppm) were higher than those in the control group (Fig. 4B). The application of iron oxide nanoparticles at concentrations of 50, 100, and 150 ppm resulted in increases of 36.05%, 76.07%, and 20.04%, respectively, compared to the control in rice roots. The fresh shoot and root weights of all treated plants showed a significant increase compared to the control group (Fig. 4C,D). The dry mass of rice shoots showed a direct relationship with the quantity of iron oxide nanoparticles when exposed to them. The dry mass increases for the 50, 100 and 150 ppm treatments were measured as 36.56%, 97.85%, and 180.65%, respectively, compared to the control (Fig. 4E). The application of nanoparticles at a concentration of 50 ppm led to a significant 101.64% enhancement in root dry mass. Similarly, a higher concentration of 100 ppm resulted in a more substantial increase of 168.85%. Under the highest dosage of 150 ppm, the root dry mass increased by 224.59% compared to the control conditions (Fig. 4F).

### Effect of IONPs on photosynthetic pigments

**Figure 5:**
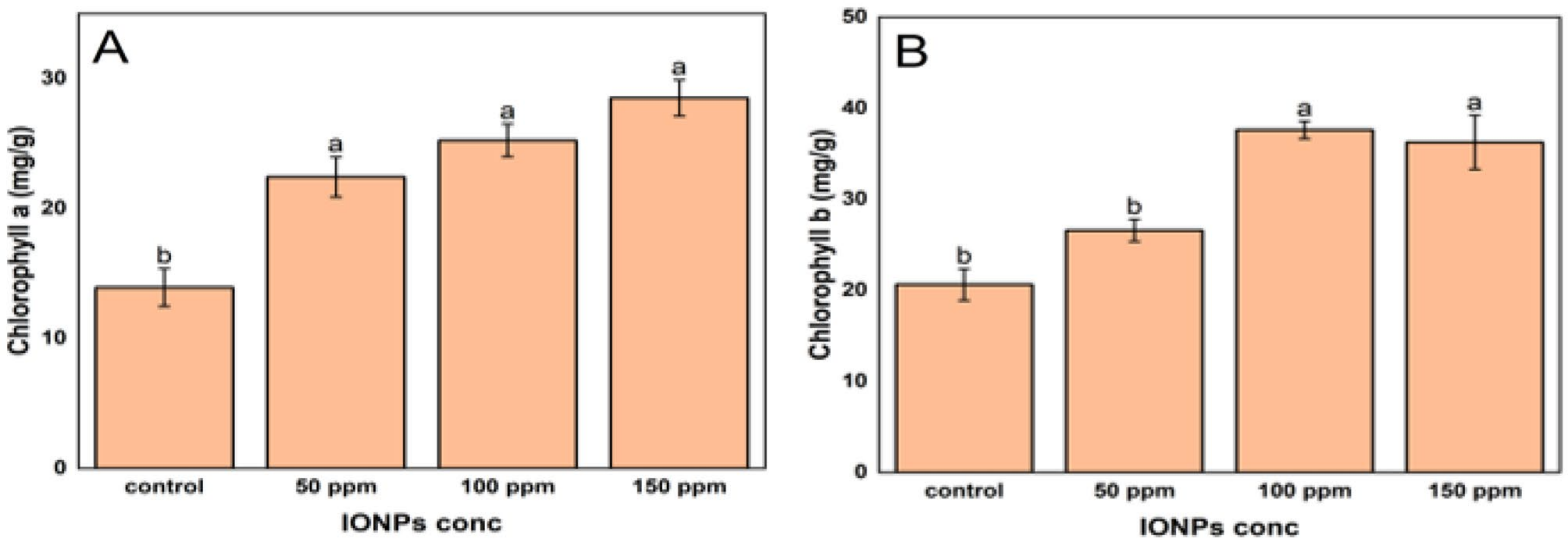
Effect of iron oxide nanoparticles on chlorophyll concentration in the leaves. (**a**) chlorophyll a content and (**b**) chlorophyll b content. Distinct letters showed significant differences at p ≤ 0.05 between different treatments. Values are means of three replicates for each treatment.

It was observed that the chlorophyll a level in the Leaf tissues for the control group was 13.93 mg/g. After treatment with nanoparticles, chlorophyll a level increased significantly. At a dose of 50 ppm of nanoparticles, chlorophyll a concentration increased by 61.10% to 22.43 mg/g from its control value of 13.93 mg/g. At a dose of 100 ppm, the chlorophyll concentration increased by 81.56% to 25.25 mg/g. At the maximal dose of 150 ppm, the final concentration of chlorophyll a was 28.51 mg/g, which is 104.59% compared to the control group (Fig. 5A). In contrast, the control group had 20.64 mg/g of chlorophyll b (Fig. 5B). Exposure to 50 ppm of nanoparticles, chlorophyll b levels increased by 28.92% to 26.59 mg/g which was increased further by 81.99% to 37.62 mg/g at a dose of 100 ppm nanoparticles. However, with an additional increase of nanoparticles, the chlorophyll b concentration was decreased. As shown in Fig. 5B, at a dose of 150 ppm of nanoparticles, chlorophyll b concentration was 36.26 mg/g, which was a 4% decrease in chlorophyll b concentration compared to 100 ppm of iron oxide nanoparticles dosage.

### Effect of NPs on grain yield and yield components

**Figure 6:**
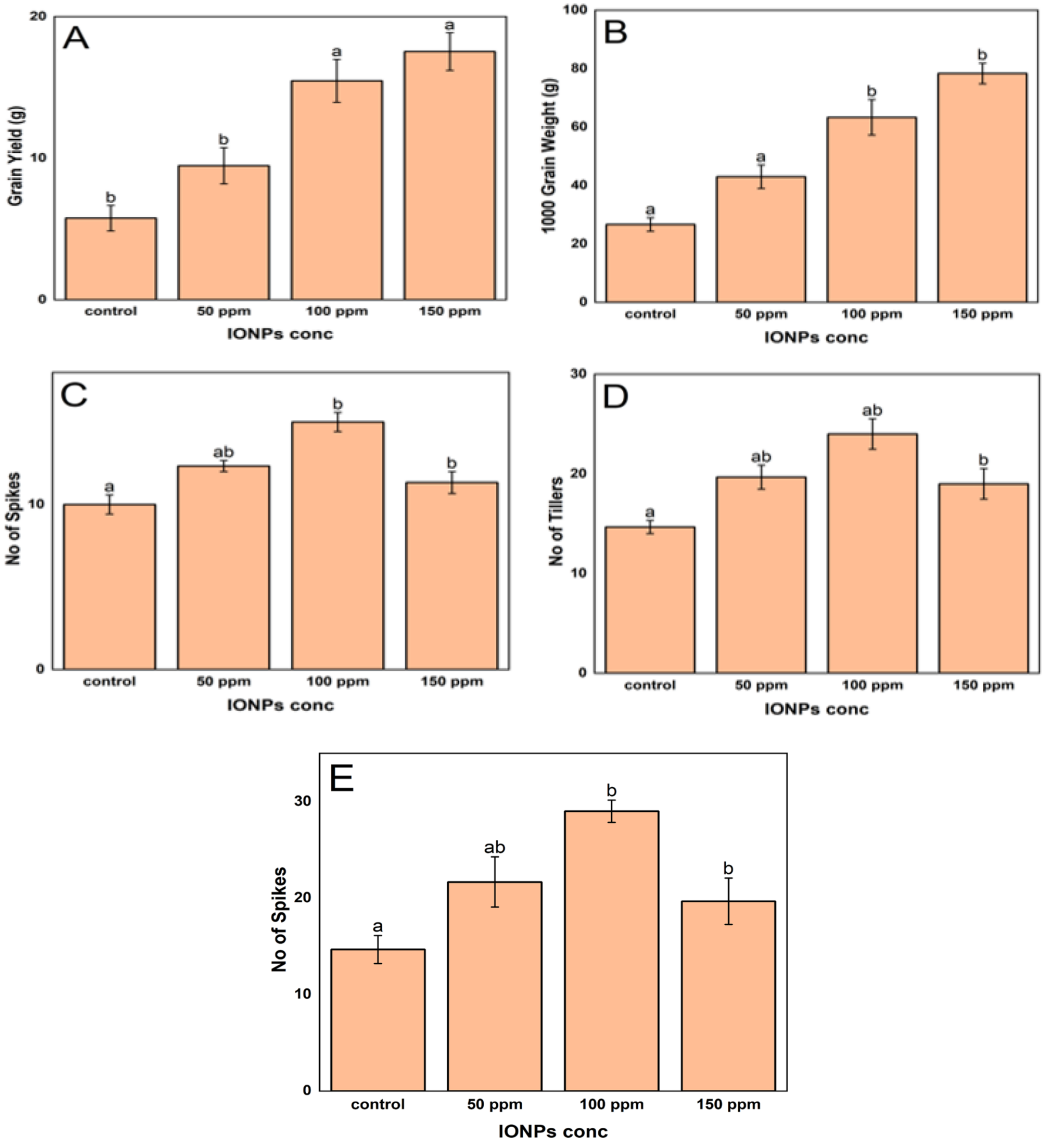
Effect of iron oxide nanoparticles on different yield components of rice. (**a**) grain yield, (**b**) grain weight (**c**) no of spikes, (**d**) no of tillers and (**e**) spike length. Distinct symbols signify significant differences at p ≤ 0.05 between different treatments. Values are means of three replicates.

The utilization of nanoparticles at different concentrations exhibited a dose-dependent improvement in specific agronomic characteristics of rice plants. The 1000-grain weight increased by 61.25%, 137.50%, and 193.75% compared to the control, when nanoparticle concentrations of 50, 100, and 150 ppm were used, respectively (Fig. 6). The length of the spike increased by 47.73% at a concentration of 50 ppm and by 97.73% at a concentration of 100 ppm. At the highest concentration of 150 ppm, there was a lower increase of 34.09%. The number of tillers increased by 23.33% at a concentration of 50 ppm and further increased by 50.00% at 100 ppm. Subsequently, there was a more modest increase of 13.33% at a concentration of 150 ppm. The grain yield showed a substantial increase, with values rising by 64.16% at 50 ppm, 168.21% at 100 ppm, and 204.05% at 150 ppm. The spike count likewise exhibited an increase, with a growth of 34.09% at a concentration of 50 ppm, 63.64% at 100 ppm, and a rise of 29.55% at 150 ppm.

### Iron content in rice grains

**Figure 7:**
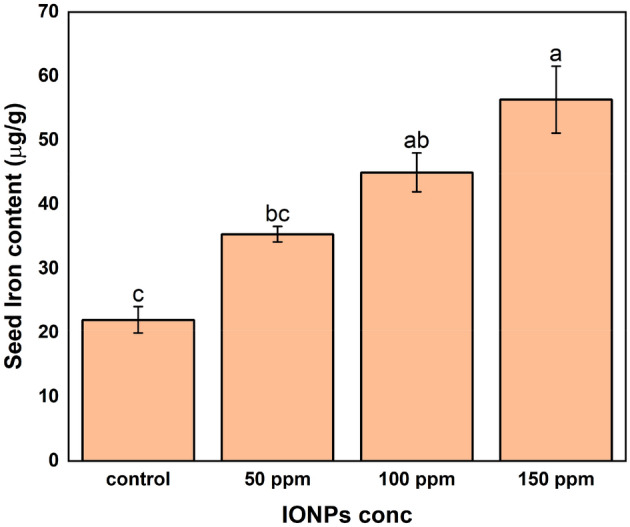
Effect of iron oxide nanoparticle dosage on the iron content of rice seeds. Different symbols designate considerable differences at p ≤ 0.05 between distinct treatments. Values are means of three replicates.

The utilization of iron oxide nanoparticles on rice seeds resulted in a substantial increase in the iron content of the seeds (Fig. 7). Compared to the untreated control, which had an average iron concentration of 22.0 ppm, the addition of iron oxide nanoparticles at a concentration of 50 ppm increased the iron content in the seeds by 60.45%, resulting in an average value of 35.3 ppm. The improvement was particularly evident when the concentration of nanoparticles reached 100 ppm. At this level, the amount of iron in the seeds increased significantly, with an average of 45.0 ppm. This represents a 104.55% increase compared to the control. Nevertheless, when the nanoparticle quantity was increased to 150 ppm, the average iron level reached 56.3 ppm, indicating a 155.91% rise. This designated that the effectiveness of the nanoparticles increases as the concentration increases.

## Discussion

**Figure 8:**
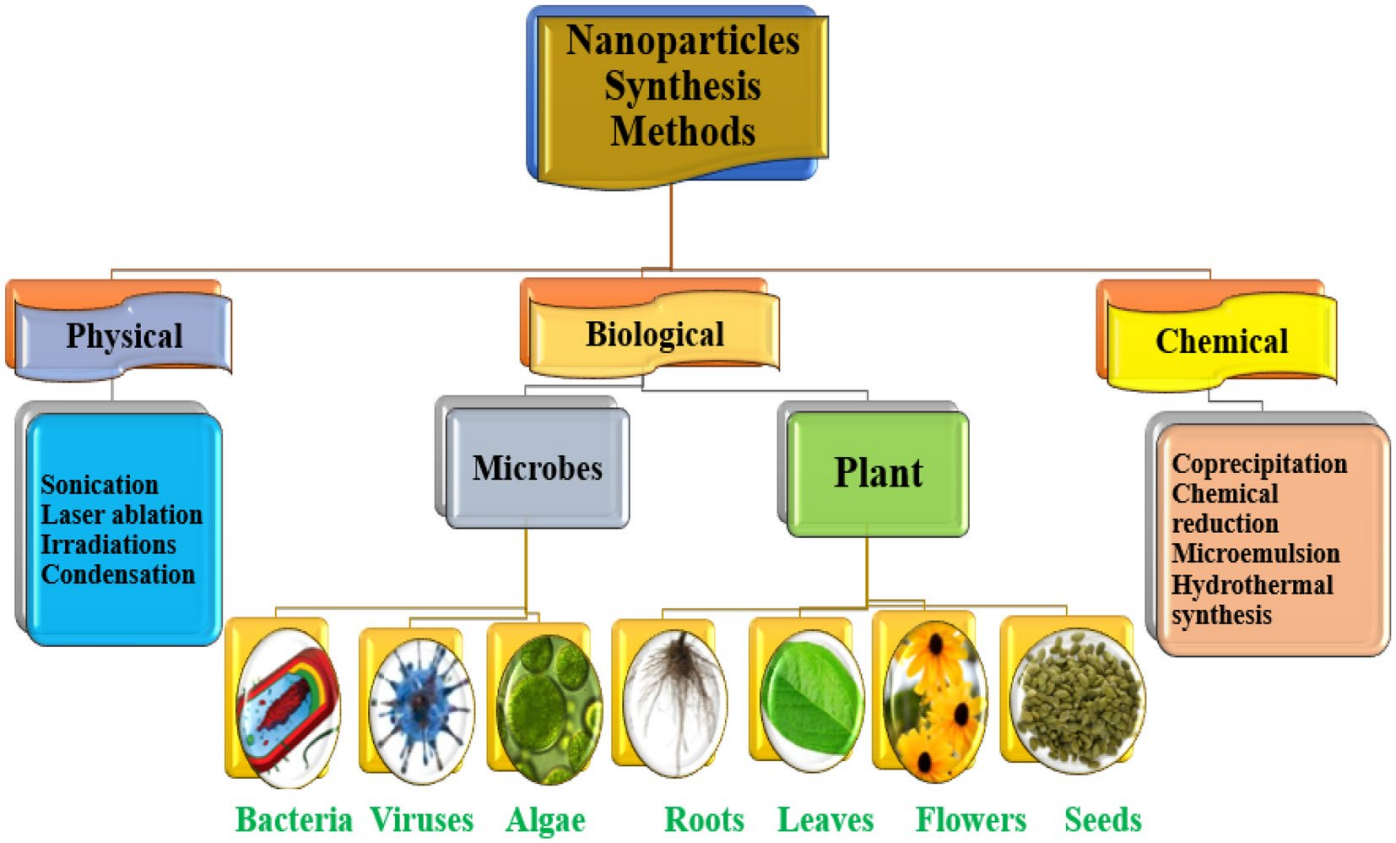
Nanoparticles synthesis methods, i.e. physical, chemical and biological.

Nanoparticles can be synthesized via three different methods: physical, chemical and biological (green synthesis) (Fig. 8). Biological method involves the use of living organisms, such as bacteria or plants, to produce nanoparticles^68,69^. Plant extracts act as stabilizing and reducing agents during plant-based synthesis, which promotes the creation of nanoparticles from a precursor salt^70^. Because it is chemical-free and safe, the biological approach has many benefits. The biological approach minimizes potential harm to the environment and living things by doing away with the necessity for dangerous chemicals and processes, in contrast to traditional chemical methods^71^. Furthermore, using plant extracts ensures that the resulting nanoparticles are biocompatible, appropriate for a range of uses, and do not endanger plant health while promoting environmental friendliness when utilized in low- and moderate concentrations^72^.

Various analytical techniques, including UV spectra (Fig. 2A), FTIR analysis, SEM, TEM, and EDX, were employed to characterize the IONPs synthesized chemically in the presence of plant extracts. UV spectra confirmed the formation of iron oxide nanoparticles, indicating a peak at 280 nm. The wavelength range 250–400 nm mostly results from ligand to metal charge transitions and partial contributions of Fe^3+^ ligand field transitions^73^. Similar spectra were also observed by^74^ and^75^ for iron oxide nanoparticles, which supports our results. The X-ray diffraction (XRD) of the synthesized IONPs reveals distinct peaks at Miller indices (311), (222), (331), and (511) together with their corresponding 2θ angles (Fig. 2B). This clearly indicates the presence of magnetite (Fe_3_O_4_) rather than hematite (α-Fe_2_O_3_). The prominent occurrence of the (311) peak, which is a distinctive feature of magnetite's cubic spinel structure, corresponds to the properties of magnetite as documented in the literature^76^. Magnetite nanoparticles having a size of 35 nm were also identified by^77,78^.

The plant extracts were subjected to FTIR analysis in order to identify the specific functional groups that are responsible for the production and stabilization of IONPs. The FTIR spectra of the plant extract exhibited distinctive peaks at 1033 cm^−1^, 1315 cm^−1^, 1424 cm^−1^, 1627 cm^−1^, 2042 cm^−1^, 2920 cm^−1^, and 3272 cm^−1^, corresponding to S=O stretching, O–H bending, N–H bending, C–H bending, and O–H stretching, respectively (Fig. 2C). Upon comparing the FTIR spectra of the synthesized IONPs (Fig. 2D), noticeable changes in the N–H bending and O–H stretching bands were detected. The shift observed indicates the participation of amine and phenolic groups derived from the plant extract in the synthesis procedure^79,80^. In particular, the peak at 1630 cm^−1^ confirmed the existence of terpenoids and alcoholics in plant extracts which helps in reducing and stabilizing the process of green synthesis^62^. The literature also presents similar findings that emphasize the significance of these functional groups in stabilizing and reducing metal ions during the creation of nanoparticles. These functional groups aid in the reduction of metal ions, resulting in the creation of iron oxide nanoparticles^79^.

The SEM and TEM images revealed that the produced IONPs had a spherical shape (Fig. 3A–D). The presence of a spherical shape is a prevalent attribute observed in well-structured nanoparticles, which aligns with the findings documented in the scientific literature on iron oxide nanoparticles^81^. Iron oxide nanoparticles having spherical agglomerated morphology were also reported by^82,83^ The estimated diameter of the nanoparticles, derived from the TEM pictures, lies within the standard nanoscale range^84^. The EDX spectra verified the elemental composition of the produced IONPs, revealing significant levels of iron (47.14%) and oxygen (16%), as detected in Fig. 2E. This composition conforms to the anticipated characteristics of iron oxide nanoparticles. The presence of elements in the EDX spectra of green manufactured IONPs that are not solely composed of iron and oxygen can be ascribed to various characteristics that are inherent to the green synthesis process. Green synthesis commonly entails the use of biological substances, such as plant extracts, which encompass a diverse range of organic chemicals and trace elements^8,31^. These biological substances serve as both reducing agents and capping and stabilizing agents during the creation of nanoparticles^85^. Consequently, components found in the plant extracts can be integrated into the nanoparticles or stick to their surface^86^.

The results of our investigation demonstrated that the levels of chlorophyll a and b exhibited a dose-dependent rise up to a certain threshold. Nevertheless, beyond this threshold, a marginal decline in the concentration of chlorophyll b was noted. The observed rise in chlorophyll levels at lower doses of IONPs (up to 100 ppm) aligns with previous studies suggesting that IONPs can function as a micronutrient for plants, promoting growth and enhancing photosynthetic activity^87^. Iron is a vital component of chlorophyll and is necessary for its synthesis^88^. The experiment revealed elevated levels of chlorophyll a and b, providing evidence that IONPs can augment the accessibility of iron in plants, resulting in an augmentation of photosynthetic pigment production^89^. This increase can also be due to metal specific-response^90^. However, at a concentration of 150 ppm, we observed a saturation point in the levels of chlorophyll a and a subsequent reduction in the levels of chlorophyll b. Excessive amounts of IONPs may induce stress responses or toxicity, perhaps leading to the degradation or suppression of chlorophyll b synthesis^91^.

The utilization of iron oxide nanoparticles greatly benefited the growth of rice plants, as seen by the noticeable rise in shoot and root lengths, as well as the improved fresh and dry biomass (Fig. 4). Recent experimental discoveries have shown that the use of IONPs has a substantial positive effect on rice growth and increased photosynthetic efficiency in rice^92^. This is likely due to their ability to decrease the toxic effects of some aspects on plants^91^. Chatterjee et al. conducted a study that showed enhanced iron uptake and increased tolerance to oxidative stress in rice plants treated with IONPs^93^. In addition, it was found that IONPs improve the antioxidant defense mechanisms in plants when they are exposed to stressful situations^94^. This suggests that IONPs may have a function in strengthening the proficiency of rice plants to withstand challenges and recover.

Moreover, the research conducted by Sebastian et al. demonstrates that carbon-bound IONPs effectively address the calcium-induced iron shortage in rice, indicating a direct influence of IONPs on rice's iron nutrition^95^. The improvement in the absorption of nutrients and the ability to handle stress is likely a significant component in the observed increase in both shoot and root growth in rice plants treated with IONPs. In addition, it was emphasized that targeted administration of small amounts of iron nanoparticles stimulates growth in settings of iron deficiency, which corresponds to the reported enhancements in growth seen in rice treated with iron nanoparticles^96^. The combined results suggest that IONPs have a diverse role in rice farming, including improving nutrient absorption, aiding in stress response, and overall enhancing plant growth^97,98^.

The results of the yield parameters of this study indicate the substantial agricultural benefits of using IONPs in rice farming. The data demonstrate that the increase in exposure of iron seeds to IONPs leads to a proportional improvement in many growth parameters. This finding supports existing research that implies nanoparticles have the potential to enhance plant growth metrics^99,100^. The administration of 100 ppm IONPs in our research resulted in a 61.25% increase in the 1000-grain weight. This discovery is consistent with the results of^101^, who observed enhanced grain weight in rice as a result of nanoparticle application. Subsequently, raising the concentration of nanoparticles to 100 and 150 ppm led to a more significant rise in grain weight, indicating a positive relationship between IONP concentration and grain weight up to a specific limit. This finding aligns with the growth enhancement caused by nanoparticles, as observed by^96^.

The length of the spikes showed a similar pattern, with the most significant rise (97.73%) observed at a concentration of 150 ppm. Nevertheless, this parameter did not exhibit a linear relationship with the highest concentration of nanoparticles. This observation aligns with the findings of^102^, who also noted that excessive nanoparticle exposure did not lead to a proportional increase in growth. This suggests the presence of a plateau effect or phytotoxicity at concentrations that exceed the optimal level. The tiller count experienced a 50% increase at a concentration of 150 ppm. This aligns with the observations made by^103^, who found that rice tillering improved as a result of increased availability of micronutrients using nanoparticle treatments. The grain yield exhibited a remarkable augmentation, especially at the maximum concentration (204.1%), indicating that the application of IONP could be efficient in improving rice yields, aligning with the findings reported by^104^. Curiously, the application of IONP led to an increase in the frequency of spikes. However, the greatest concentration of IONP resulted in a minor increase compared to the treatment with a concentration of 100 ppm.

Recent studies on the use of IONPs on rice seeds have revealed their substantial contribution to increasing the iron content in these seeds through various processes. Reference^96^ research revealed that lower concentrations of FeO nanoparticles have a dual effect on rice growth and grain quality. They enhance many metrics, such as height, pigment content, and dry weight of grains, while also enriching the microbial population in the rhizosphere. This process of microbial enrichment is believed to enhance the accessibility and absorption of iron in rice seeds. Simultaneously, the research conducted by^105^ showed that FeO nanoparticles, when compared to hydropriming, increase the iron concentration in rice seeds. The fundamental mechanism, although not precisely explained, relies on enhanced iron absorption and integration.

In addition, Feng et al. discovered that Fe3O4 nanoparticles increase the iron concentration in rice seeds and also improve photosynthetic efficiency, as well as the accessibility of vital nutrients such as iron and phosphorus^106^. This indicates the presence of a process that involves efficient absorption and dispersion of these nanoparticles. A study on wheat reveals that treating it with iron oxide nanoparticles resulted in higher iron levels in the shoot. This rise can be linked to improvements in photosynthetic characteristics, antioxidant balance, and nutrient availability^101^. These findings suggest that there may be comparable mechanisms at work in rice seeds.

The study performed by Zhang et al. demonstrated that the presence of graphene oxide nanoparticles in rice seeds led to an increase in iron concentration^107^. This was achieved by promoting the movement and concentration of iron in the shoots, which was facilitated by acidification of the nutrient solution and subsequent mobilization of iron. Collectively, these studies emphasized that iron oxide nanoparticles have a significant influence on the iron levels in rice seeds. The mechanisms encompass enhanced absorption and use of nutrients, alterations in the dynamics of microorganisms in the rhizosphere, increased photosynthetic and antioxidant activities, and mitigation of stress.

**Figure 9:**
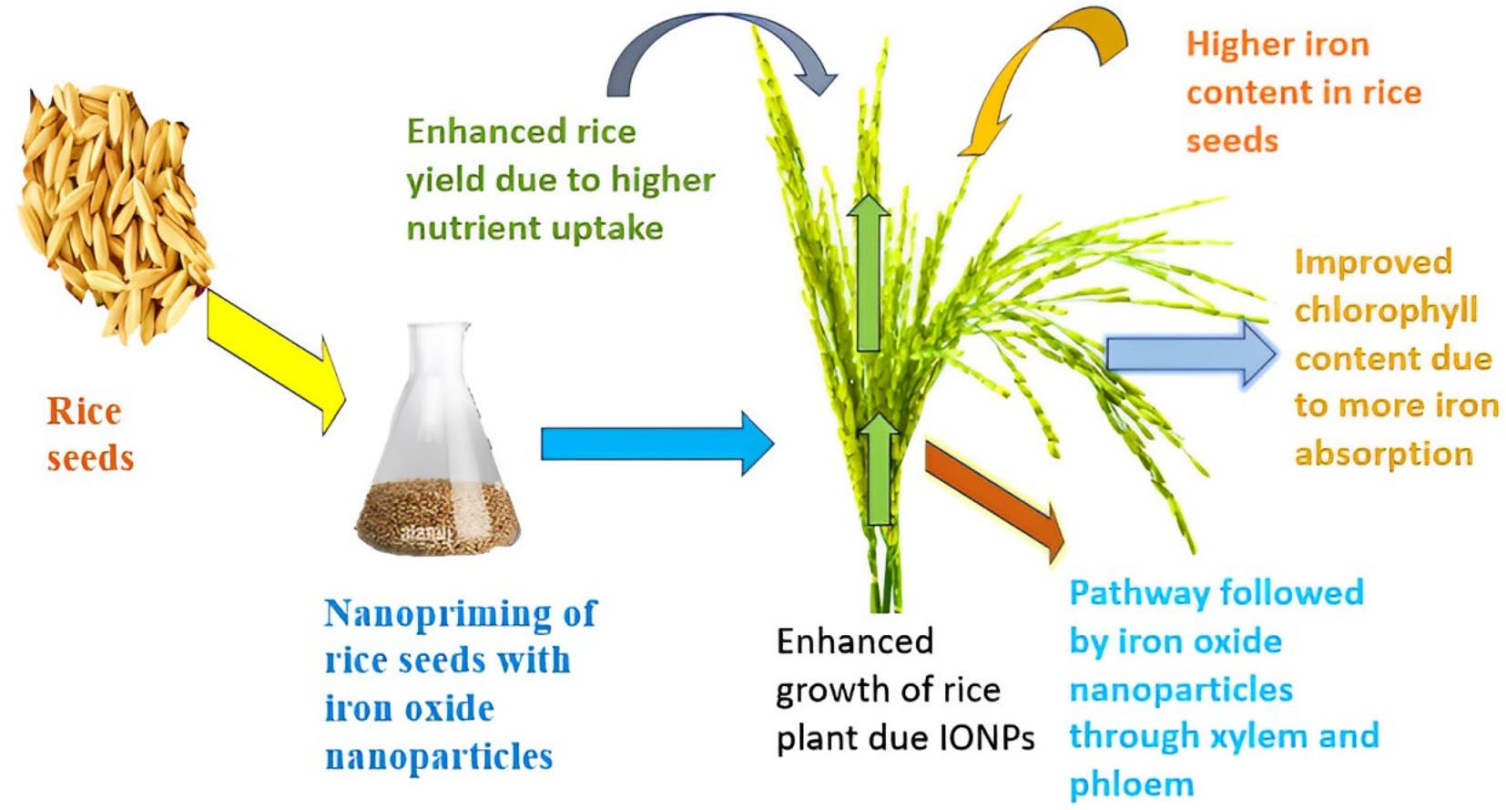
Impact of rice seeds nanopriming on the growth of rice plants and iron biofortification in seeds.

Nanopriming using IONPs generated from green sources has the potential to improve plant development and increase iron levels^108^. This is achieved through a complex system that operates at both the cellular and molecular levels. These nanoparticles possess distinctive physicochemical features as a result of their tiny size at the nanoscale, allowing them to efficiently penetrate and distribute within plant tissues^109^. Once assimilated, IONPs are hypothesized to function as catalysts, enabling several biochemical and physiological activities^110^. This encompasses heightened iron absorption, which is vital for the production of chlorophyll and the process of photosynthesis, resulting in enhanced development and vitality^111^. In addition, IONPs can stimulate the increased expression of particular genes associated to the transportation and metabolism of iron, hence enhancing the absorption and use of iron^112^. Furthermore, their function in stimulating antioxidant defense mechanisms aids in reducing oxidative stress, which is frequently linked to insufficient nutritional levels^16^. The simultaneous function of these nanoparticles in enhancing iron feeding and strengthening the plant's stress response mechanisms explains the observed enhancements in growth and iron content in rice plants treated with them. The possible reasons how can rice seeds nanopriming affect growth of rice plants and iron biofortification is shown in (Fig. 9).

## Conclusion

This study confirms that green synthesized IONPs using *G. glabra* plant extracts significantly enhanced both the development and iron content of rice plants. The generated IONPs were proven to have a spherical form using SEM and TEM analysis. Nanopriming using IONPs significantly extended the lengths of both shoots and roots, particularly at doses of 50 and 100 ppm. This treatment also increased biomass, as indicated by the improved dry mass. The agronomic characteristics, such as 1000-grain weight, the length of the spike, the number of tillers, and the yield of grains, showed considerable improvement. This demonstrates that IONPs play a role in increasing crop productivity in a manner that depends on the dosage. Furthermore, the addition of IONPs at concentrations of 50 and 100 ppm significantly improved the biofortification of iron in rice grains. However, the effectiveness of the treatment reached a maximum level at a concentration of 150 ppm. These findings have practical consequences in the field of agricultural biofortification, providing an environmentally practical approach to enhance crop productivity and nutritional value. The widespread use of these nano-priming techniques has the potential to be applied to other crops, resulting in significant improvements in global food systems.

### Future perspectives

Long-term environmental and safety effects of IONP treatments in rice agriculture should be studied. Optimizing nanoparticle applications requires understanding IONP-induced growth and nutrient uptake increases. IONP efficacy must be assessed across environmental conditions and rice genotypes to ensure generalizability. Field experiments are needed to demonstrate IONP's viability and scalability. IONP treatments combined with other agronomic tactics may improve crop yields and nutrition.

## Data Availability

Data will be made available upon request by corresponding author Mian Afaq Ahmad.
